# Expression and diagnostic value of lncRNA MALAT1 and NLRP3 in lower limb atherosclerosis in diabetes

**DOI:** 10.1186/s12902-024-01557-w

**Published:** 2024-03-04

**Authors:** Juan Li, Chun Wang, Chen Shao, Jiaxin Xu

**Affiliations:** 1 Department of Endocrinology, The Second Affiliated Hospital of Bengbu Medical University, 233040 Bengbu, Anhui China; 2 Department of General Medicine, The Second Affiliated Hospital of Bengbu Medical University, 233040 Bengbu, Anhui China; 3 Department of Pediatrics, The First Affiliated Hospital of Bengbu Medical University, 233004 Bengbu Anhui, China

**Keywords:** lncRNA MALAT1, Type 2 diabetes mellitus, Lower extremity atherosclerosis, NLRP3

## Abstract

**Objective:**

This study aimed to examine the diagnostic predictive value of long non-coding RNA (lncRNA) metastasis-associated lung adenocarcinoma transcript 1(MALAT1) and NOD-like receptor protein 3(NLRP3) expression in patients with type 2 diabetes mellitus(T2DM) and lower extremity atherosclerosis disease (LEAD).

**Methods:**

A total of 162 T2DM patients were divided into T2DM with LEAD group (T2DM + LEAD group) and T2DM alone group (T2DM group). The lncRNA MALAT1 and NLRP3 expression levels were measured in peripheral blood, and their correlation was examined. Least absolute shrinkage and selection operator (LASSO) regression model was used to screen for the best predictors of LEAD, and multivariate logistic regression was used to establish a predictive model and construct the nomogram. The effectiveness of the nomogram was assessed using the receiver operating characteristic (ROC) curve, area under the curve (AUC), calibration curve, and decision curve analysis (DCA).

**Results:**

The levels of the lncRNA MALAT1 and NLRP3 in the T2DM + LEAD group were significantly greater than those in the T2DM group (*P* <0.001), and the level of the lncRNA MALAT1 was positively correlated with that of NLRP3 (*r* = 0.453, *P*<0.001). The results of the LASSO combined with the logistic regression analysis showed that age, smoking, systolic blood pressure (SBP), NLRP3, and MALAT1 were the influencing factors of T2DM with LEAD(*P*<0.05). ROC curve analysis comparison: The discriminatory ability of the model (AUC = 0.898), MALAT1 (AUC = 0.804), and NLRP3 (AUC = 0.794) was greater than that of the other indicators, and the predictive value of the model was the greatest. Calibration curve: The nomogram model was consistent in predicting the occurrence of LEAD in patients with T2DM (Cindex = 0.898). Decision curve: The net benefit rates obtained from using the predictive models for clinical intervention decision-making were greater than those obtained from using the individual factors within the model.

**Conclusion:**

MALAT1 and NLRP3 expression increased significantly in T2DM patients with LEAD, while revealing the correlation between MALAT1 and NLRP3. The lncRNA MALAT1 was found as a potential biomarker for T2DM with LEAD.

**Supplementary Information:**

The online version contains supplementary material available at 10.1186/s12902-024-01557-w.

## Introduction

In recent years, the prevalence of diabetes has been increasing annually, and the latest data show [1] that more than half a billion people have diabetes around the world. China has become one of the countries with the largest number of adults with diabetes mellitus. Atherosclerosis (AS) is a chronic inflammatory reaction of arterial endothelial injury [2]. Vascular endothelial cell (EC) dysfunction is the initial process that induces the formation of atherosclerotic plaques. Long-term hyperglycemia can lead to the release of large amounts of cellular inflammatory factors, inflammatory damage, vascular endothelial cell dysfunction, and atherosclerotic plaque formation. Hyperglycemia can accelerate the progression of atherosclerosis. The important pathogenesis of diabetes is insulin resistance. C-peptide is now considered a potential biomarker of insulin resistance in type 2 diabetes mellitus(T2DM). It has been reported that [3] C-peptide has anti-inflammatory and anti-atherosclerotic effects in type 1 diabetes and early type 2 diabetes, and plays a promoting role in the pathogenesis of atherosclerosis in late T2DM.There is still a contradiction between diabetes and atherosclerosis, and the specific pathogenesis is still unclear. Lower extremity atherosclerotic disease (LEAD) is a common chronic complication of diabetes. It can lead to severe ischemia, ulcers, necrosis, and amputation of the lower extremity in the later stage because of the lack of typical clinical manifestations in the early stage, and the disability and mortality rates increase significantly [4]. At present, there is no specific biomarker for atherosclerosis of the lower limbs in patients with diabetes. Thus, it is extremely important to find specific biomarkers to screen patients at high risk for LEAD and for early diagnosis and control of disease progression in asymptomatic diabetic patients.

Long-noncoding RNA (lncRNA) is defined as RNA molecules with a length greater than 200 nucleotides that are not translated into proteins. It is a significant regulatory factor in various biological processes and is being developed into a new biomarker and therapeutic target for various diseases. LncRNA metastasis-associated lung adenocarcinoma transcript 1 (MALAT1) which is related to inflammation, oxidative stress, and angiogenesis, is widely expressed in a variety of tissues in vivo and has been confirmed to play a significant role in the complications of diabetes [5]. Many studies have discovered that MALAT1 expression is increased in human umbilical vein endothelial cells incubated in high glucose; overexpression of MALAT1 promotes an inflammatory response in a mouse model of AS, thereby contributing to the pathogenesis of AS [6, 7]. The latest studies have shown that MALAT1 expression levels are significantly greater in patients with coronary artery disease (CAD) and T2DM, and can be used as a better diagnostic indicator for CAD [8]. Activation of NOD-like receptor protein 3 (NLRP3) is strongly associated with metabolic inflammatory diseases such as diabetes, atherosclerosis, and obesity. It is found that the expression of NLRP3 inflammasome in diabetes patients with lower limb arterial disease is significantly increased, which may participate in the pathological progress of the disease [9]. In vivo and vitro studies have shown [10] that high glucose induces activation of the NLRP3 inflammasome in endothelial cells, triggering inflammatory reactions and pyroptosis, leading to endothelial cell dysfunction. The NLRP3 inflammasome is strongly linked between diabetes and AS. Studies have shown that lncRNA MALAT1 can regulate the expression of NLRP3-related genes [11, 12]. However, it has not been reported whether lncRNA MALAT1 may participate in the pathogenesis of lower limb atherosclerosis in diabetes through NLRP3-mediated pyroptosis. The study analyzed the expression of lncRNA MALAT1 and NLRP3 in lower limb atherosclerotic disease in diabetes patients, explored the relationship between them and the disease, and whether lncRNA MALAT1 may be a new biomarker of LEAD. To provide a reliable basis for predicting the development of LEAD in T2DM patients.

## Materials and methods

### Research objects

A total of 162 T2DM patients hospitalized in the Department of Endocrinology and General Department of the Second Affiliated Hospital of Bengbu Medical University from October 2022 to October 2023 were enrolled, including 64 cases of type 2 diabetes mellitus alone (T2DM group) and 98 cases of type 2 diabetes mellitus with lower limb atherosclerosis disease (T2DM + LEAD group). T2DM was diagnosed according to the China Diabetes Prevention and Treatment Guidelines (2020): diabetes symptoms (polyuria, polydipsia, polydipsia, and weight loss) and fasting blood glucose (FBG) level ≥ 7.0 mmol/L, random blood glucose level ≥ 11.1 mmol/L, or blood glucose level ≥ 11.1 mmol/L in the two-hour 75 g oral glucose tolerance test. Patients included in the LEAD group include those with thickening of the intima-media layer and plaque formation in the lower limb arteries. Exclusion criteria were as follows: diabetes ketoacidosis, hypertonic hyperglycemic syndrome, hypoglycemic coma, diabetes nephropathy, diabetes retinopathy, severe liver and kidney function damage, gout, Alzheimer’s disease, acute symptomatic stroke, Parkinson’s disease, neurodegenerative diseases, acute myocardial infarction, tumors, and inflammatory diseases such as bacterial and viral infections. All study subjects were required to sign informed consent, which was subjected to approval by the Ethics Committee.

### Clinical data collection

At the time of admission, basic information about the patients was collected, including sex, age, duration, history of smoking, history of drinking, height, weight, body–mass index (BMI), systolic blood pressure (SBP), diastolic blood pressure (DBP), history of previous illnesses (Hypertension), and history of medication. Fasting blood glucose (FBG), glycated hemoglobin (HbA1C), triglycerides (TG), total cholesterol (TC), high-density lipoprotein cholesterol (HDL), low-density lipoprotein cholesterol (LDL), insulin, uric acid (UA), and creatinine (Cr) were detected. The formula for evaluating a patient’s insulin resistance using the insulin resistance index (HOMA-IR) is HOMA-IR = FBG * fasting insulin/22.5.

### Lower limb arterial ultrasound

The study subjects were placed in the supine position, and ultrasound equipment ((ie33, Philips Ultrasound Imaging Systems, Shanghai) was used to scan bilateral common femoral arteries, superficial femoral arteries, popliteal arteries, and anterior and posterior tibial arteries to measure arterial intima-media thickness (IMT). The number, echo, shape, and size of the plaques were observed and recorded. Participants with an IMT < 1.0 mm were defined as normal, whereas those with 1.0 mm ≤ IMT ≤ 1.5 mm were defined as thickening. Compared with the surrounding tube wall, the thickness of the intermedia increased by 50% or increased by 0.5 mm. A plaque was defined as a thickened local artery wall ≥ 1.5 mm. In this study, the atherosclerotic lesions in the lower extremity were intima thickening and plaque formation.

### Measurement of lncRNA MALAT1 and NLRP3

The expression of MALAT1 was detected by real-time fluorescence quantitative reverse transcription polymerase chain reaction (qRT-PCR). Total RNA was extracted according to the Trizol instructions (Invitrogen, CA, USA), and the extracted RNA was reverse transcribed into cDNA according to the kit instructions (Invitrogen, CA, USA). The cDNA was used as the template, and upstream and downstream primers were used for amplification. GAPDH was used as the internal control, 3 compound pores were set in each sample, and the relative quantification of mRNA was calculated using the mean of 2^− ΔΔCt^. The sequences of the primers were as follows: MALAT1: Forward, 5’- GGGGCTCAGTTGCGTAATGG-3’ and Reverse, 5’- AACACCTCACAAAACCCCCG-3’; GADPH: Forward, 5’-AGGTCGGAGTCAACGGATTT-3’ and Reverse, 5’-GACGGTGCCATGGAATTTGC- 3’. Serum NLRP3 levels were measured by enzyme-linked immunosorbent assay (ELISA) by kit instructions (Jingmei Biotech, Beijing, China).

### Statistical analysis

The data were analyzed using SPSS 20.0. Normally distributed variables were expressed as mean ± standard deviation and analyzed using the t-test, nonnormally distributed variables were expressed as median P50 (P25, P75) and analyzed using the Mann-Whitney U-test. Count data were expressed as the number of cases or percentages and were analyzed using the chi-square test. Pearson or Spearman correlation was used for correlation analysis. Using R4.3.1 statistical analysis software, Lasso regression model was used to exclude collinear variables and screen for predictive factors of LEAD occurrence. Multivariate logistic regression was used to analyze the independent risk factors. A nomogram was made to visualize the model, receiver operating characteristic curve (ROC), area under the curve (AUC), and calibration curve were made to evaluate the model differentiation and calibration. Decision curve analysis (DCA) was drawn to evaluate the clinical net benefit. A value of *P* < 0.05 was considered statistically significant.

## Results

### Basic characteristics of subjects

The age and duration of disease in the T2DM + LEAD group were significantly greater than those in the T2DM group (*P* < 0.001), while the systolic blood pressure and insulin resistance index were greater than those in the T2DM group (*P* < 0.05). There were no statistically significant differences in gender, smoking, drinking, hypertension history, drug use (hypoglycemic drugs, hypertension drugs, statins), BMI, DBP, FBG, HbA1C, TC, TG, LDL, HDL, UA, and Cr between the two groups(*P* > 0.05)(Table 1).

**Table 1:** Comparison of clinical data between two groups

	T2DM (*n* = 64)	T2DM + LEAD (*n* = 98)	P value
Male (%)	51 (79.69%)	80 (81.63%)	0.758
Age (year)	51 (45,58)	59 (52,68)	<0.001*
Duration (year)	3.00 (0.31,7.00)	6.00 (1.88,10.00)	<0.001*
Smoking (%)	23 (35.94%)	45 (45.92%)	0.208
Drinking (%)	31 (48.44%)	49 (50.00%)	0.846
Hypertension (%)	26 (40.63%)	55 (56.12%)	0.054
Hypoglycemic drugs (%)	47 (73.44%)	84 (85.71%)	0.052
Hypertension drugs (%)	25 (39.06%)	51 (52.04%)	0.106
Statin drugs (%)	3 (4.69%)	12 (12.24%)	0.105
BMI (kg/m^2^)	24.82 (23.28,27.40)	24.30 (22.99,27.07)	0.301
SBP (mmHg)	126.00 (117.00,144.50)	135.00 (121.00,150.25)	0.040*
DBP (mmHg)	85.25 ± 10.60	85.19 ± 11.29	0.975
FBG (mmol/L)	8.22 (6.94,10.74)	7.81 (6.55,10.95)	0.742
HOMA-IR	2.35 (1.80,4.11)	3.05 (2.08,4.60)	0.045*
HbA1C (%)	8.75 (7.43,10.70)	8.95 (7.30,10.63)	0.915
TG (mmol/L)	2.06 (1.59,2.86)	1.71 (1.18,2.84)	0.095
TC (mmol/L)	4.89 (4.33,5.31)	4.81 (4.15,5.54)	0.997
LDL (mmol/L)	2.72 ± 0.63	2.73 ± 0.89	0.967
HDL (mmol/L)	1.28 (1.15,1.61)	1.30 (1.12,1.49)	0.342
UA (umol/L)	298.81 ± 67.62	290.86 ± 74.10	0.490
Cr (umol/L)	61.33 ± 13.40	61.39 ± 14.46	0.979

Values are presented as median (range), number (%), or mean ± SD deviation, *P < 0.05 vs. T2DM group

BMI: body mass index; SBP: systolic blood pressure; DBP: diastolic blood pressure; FBG: fasting blood glucose; HbA1c: hemoglobin A1c; TG: triglyceride; TC: total cholesterol; LDL: low-density lipoprotein; HDL: high-density lipoprotein; UA: uric acid; Cr: creatinine

### Comparison of the lncRNAMALAT1 and NLRP3 expression between two groups

**Fig. 1:**
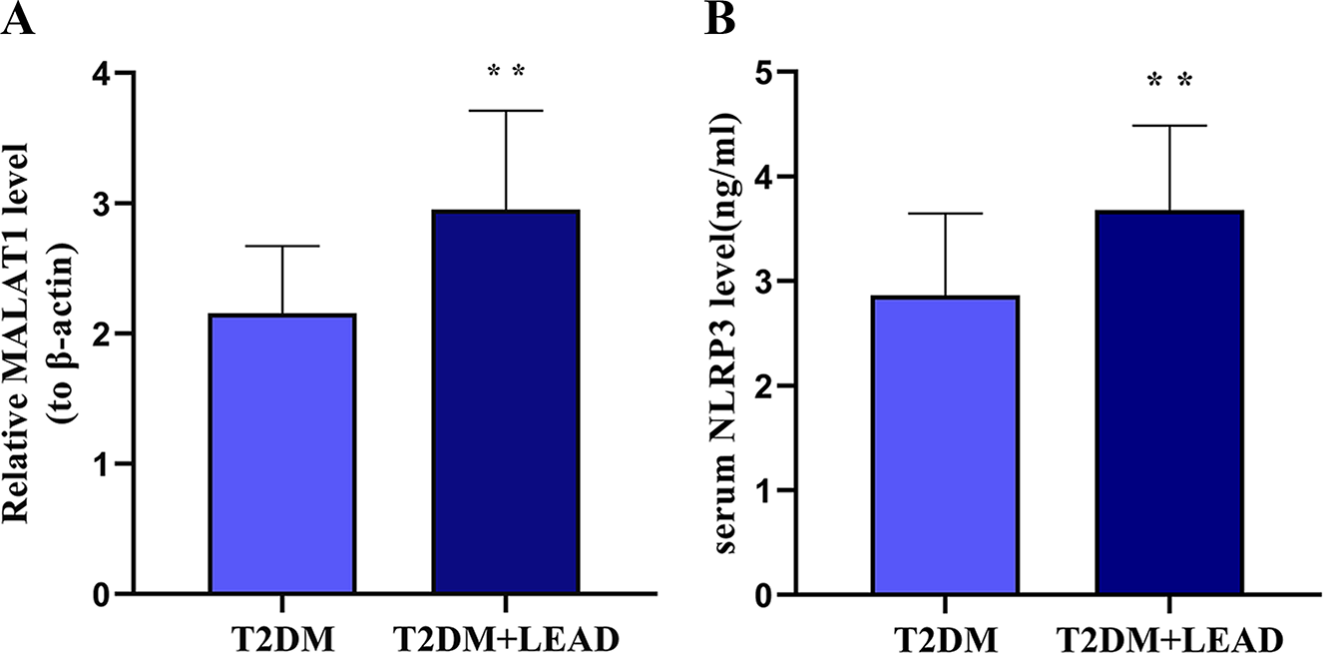
Expression of lncRNA MALAT1 and NLRP3 in two groups.(**A**) The lncRNA MALAT1 expression in patients in two groups. (**B**) Serum NLRP3 expression in patients in two groups. Compared with the T2DM group, **P < 0.01.

As shown in Fig. 1, compared with that in the T2DM group, the expression level of the lncRNA MALAT1 was significantly greater in the T2DM + LEAD group. The NLRP3 concentration in the T2DM + LEAD group was significantly greater than that in the T2DM group, and the difference was statistically significant (*P* < 0.001).

### Correlation analysis of lncRNA MALAT1 and NLRP3

**Fig. 2:**
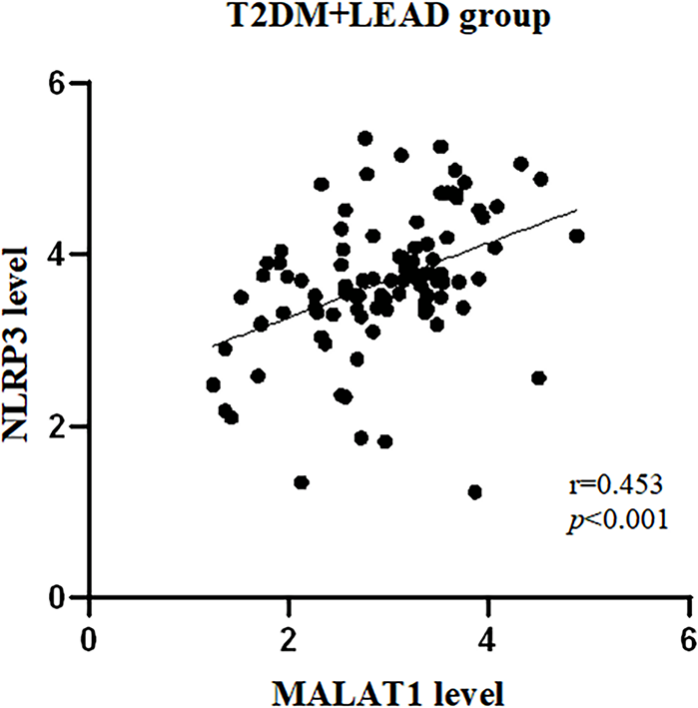
Correlations between lncRNA MALAT1 and NLRP. The expression level of the lncRNA MALAT1 was positively correlated with that of NLRP3.

Correlations between lncRNA MALAT1 and NLRP3 in patients with T2DM combined with LEAD were analyzed by Spearman’s method. The results showed that the expression level of lncRNA MALAT1 was positively correlated with NLRP3 concentration, and with the correlation coefficients of *r* = 0.453(*P*<0.001) (Fig. 2).

### Least absolute shrinkage and selection operator (LASSO) regression filters the independent variables

**Fig. 3:**
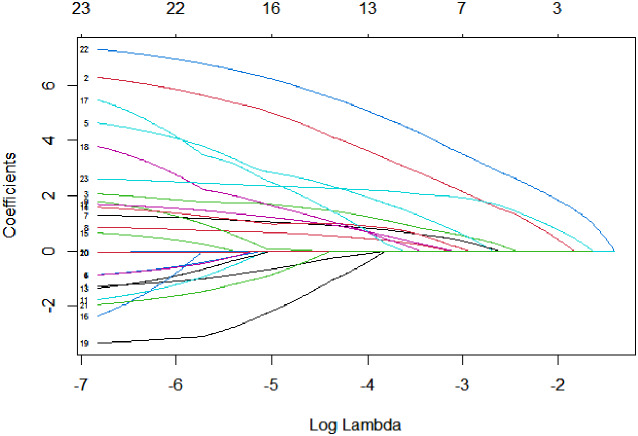
LASSO regression pathway. Variable selection using LASSO logistic regression yields coefficient profiles for 23 variables. As the penalty coefficient λ increases, the coefficients of more and more variables are compressed until they are compressed to 0.

**Fig. 4:**
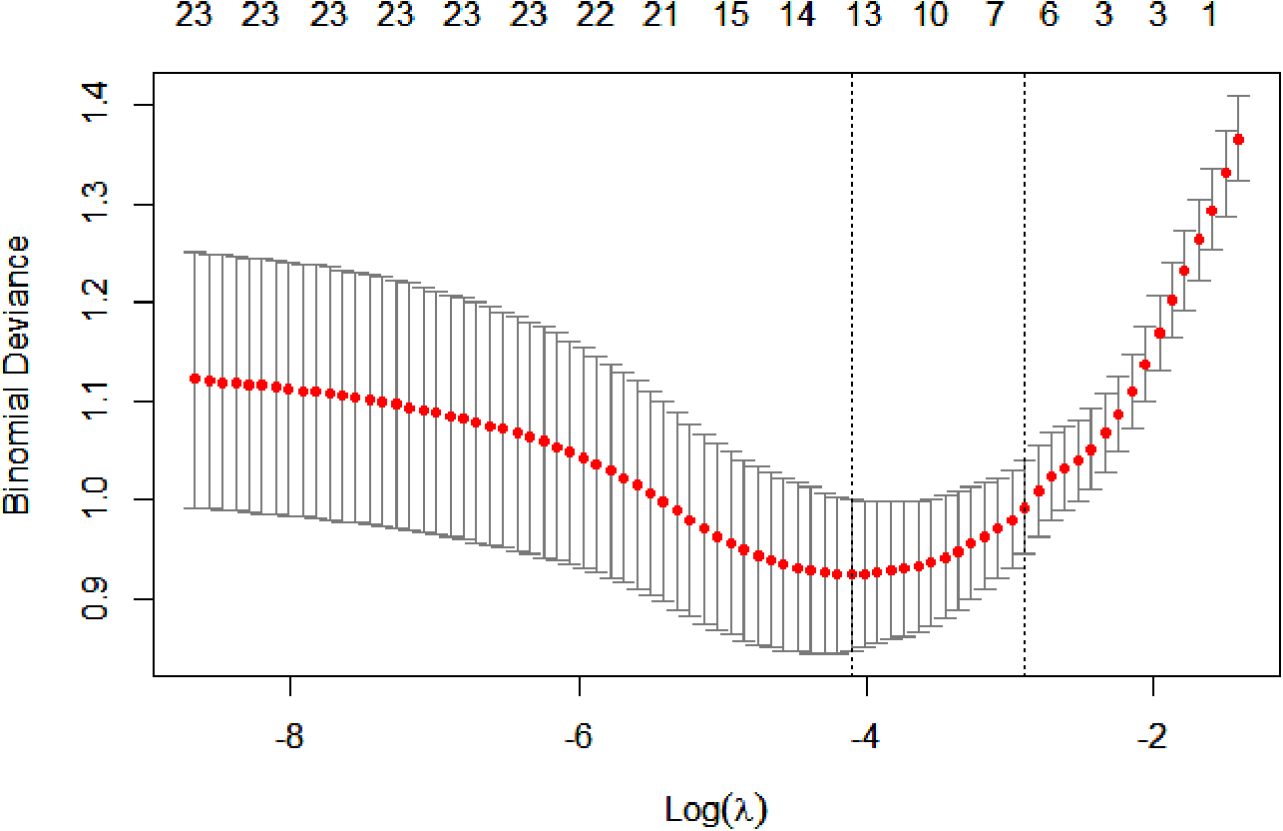
Cross-validation of LASSO regression. The best penalty coefficient lambda was selected using a twentyfold cross-validation and minimization criterion. The graph has log(lambda) in the horizontal coordinate, binomial deviance in the vertical coordinate, and vertical dashed lines plotted against one standard error criterion. Six variables with nonzero coefficients were selected by optimal lambda.

We included gender, age, duration, smoking history, alcohol consumption history, history of hypertension, medication use (hypoglycemic drugs, antihypertensive drugs, statins), BMI, SBP, DBP, FBG, HOMA-IR, HbA1C, TC, TG, LDL, HDL, UA, Cr, NLRP3, MALAT1 as independent variables and the occurrence of LEAD as the dependent variable in the LASSO regression model for variable screening. The results suggested that the optimal model was obtained when the penalty coefficient was λ = 0.055, and there were six indicators of the selected variables, which were age, duration, smoking, SBP, NLRP3, and MALAT1 (Figs. 3 and 4).

### Logistic regression analysis to establish a nomogram model

**Fig. 5:**
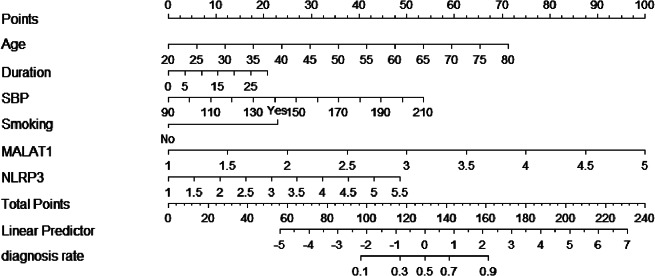
Nomogram of LEAD prediction model. The corresponding values of each variable are scored, and the total score is then obtained by summing the scores of all variables, and a vertical line plotted downward from the total score can be labeled to indicate the estimated probability of LEAD occurring in a patient with T2DM.

A multifactorial logistic regression model was constructed with the six variables selected by LASSO regression as independent variables and the occurrence of LEAD as the dependent variable. The results showed that age, smoking, SBP, NLRP3, and MALAT1 were independent risk factors for LEAD in patients with type 2 diabetes (*P* < 0.05) (Table 2). The above risk factors were used as predictors to construct a nomogram model for predicting type 2 diabetes combined with LEAD (Fig. 5).

**Table 2:** Multivariate regression equation parameters of LEAD prediction model

Variable	β	SE	Wald	OR	95%CI	P value
Age	0.082	0.026	3.190	1.085	1.035–1.144	0.014
Duration	0.048	0.048	0.990	1.049	0.957–1.157	0.320
SBP	0.031	0.013	2.280	1.031	1.006–1.060	0.023
Smoking	1.570	0.503	3.120	4.806	1.862–13.584	0.002
MALAT1	1.715	0.394	4.350	5.554	2.690-12.767	< 0.001
NLRP3	0.741	0.295	2.520	2.098	1.192–3.814	0.012

SBP: systolic blood pressure; β, regression coefficient; SE, standard error; Wald, chi‑square value; OR, risk value; CI, confidence interval

### Validation of the nomogram for predicting the risk of T2DM in patients with LEAD

**Fig. 6:**
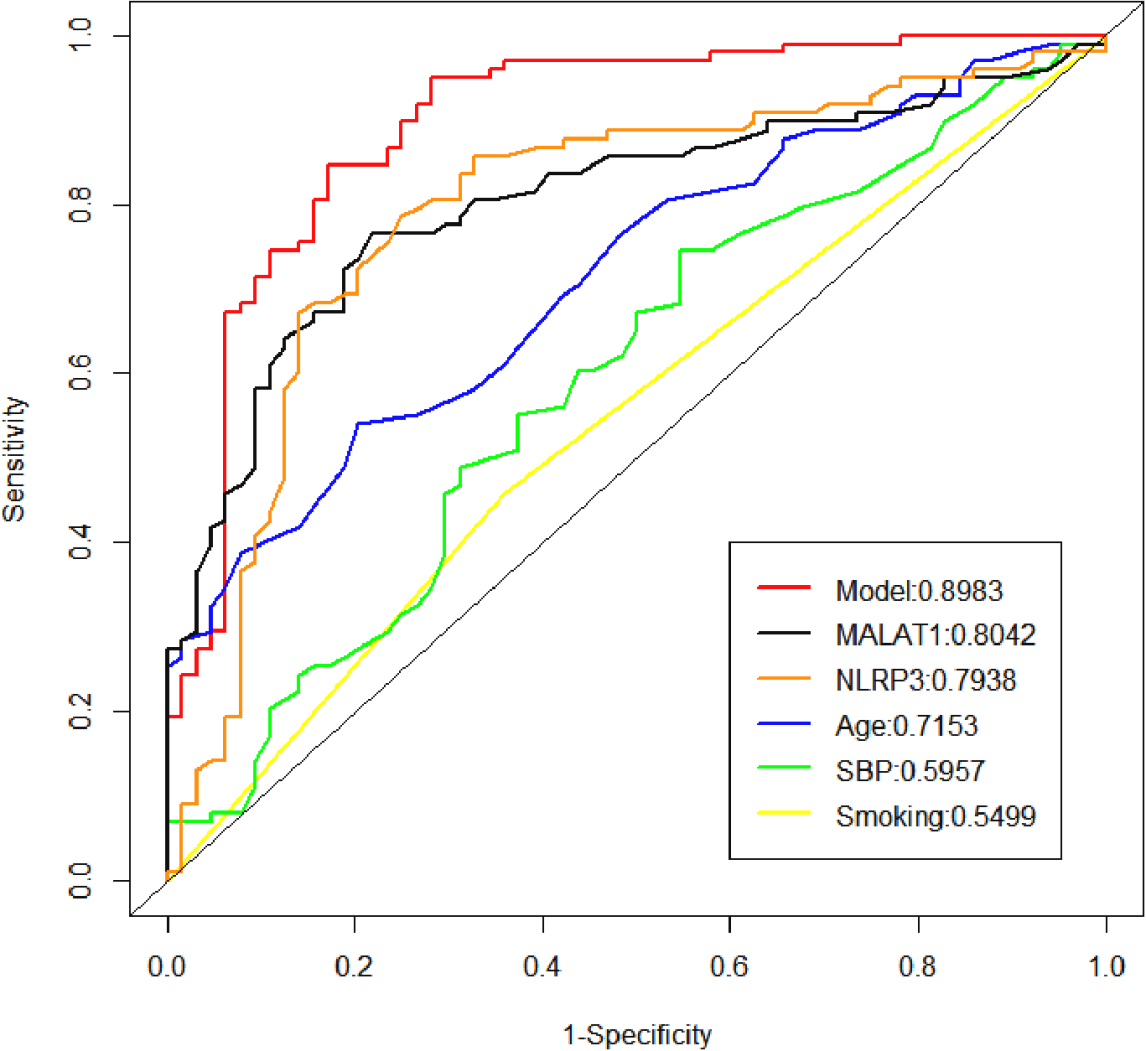
ROC analysis of the laboratory parameters and model

The ROC curve was used to evaluate the model’s discrimination AUC = 0.898 (95% CI = 0.847–0.950), and the best cut-off point was achieved with Jordon’s index of 1.685 (sensitivity 88.8%, specificity 79.7%). ROC analysis was also used to compare the predictive value of individual parameter indicators in the model, and the AUC of MALAT1 and NLRP3 for predicting combined LEAD in T2DM patients were 0.804 and 0.794, which were greater than those of age (AUC = 0.715), SBP (AUC = 0.596), and smoking (AUC = 0.550). Compared with MALAT1, which has the highest predictive value for a single indicator parameter, the model has a higher predictive value (AUC = 0.898 vs. 0.804), indicating that the model has a better predictive ability (Fig. 6; Table 3).

**Fig. 7:**
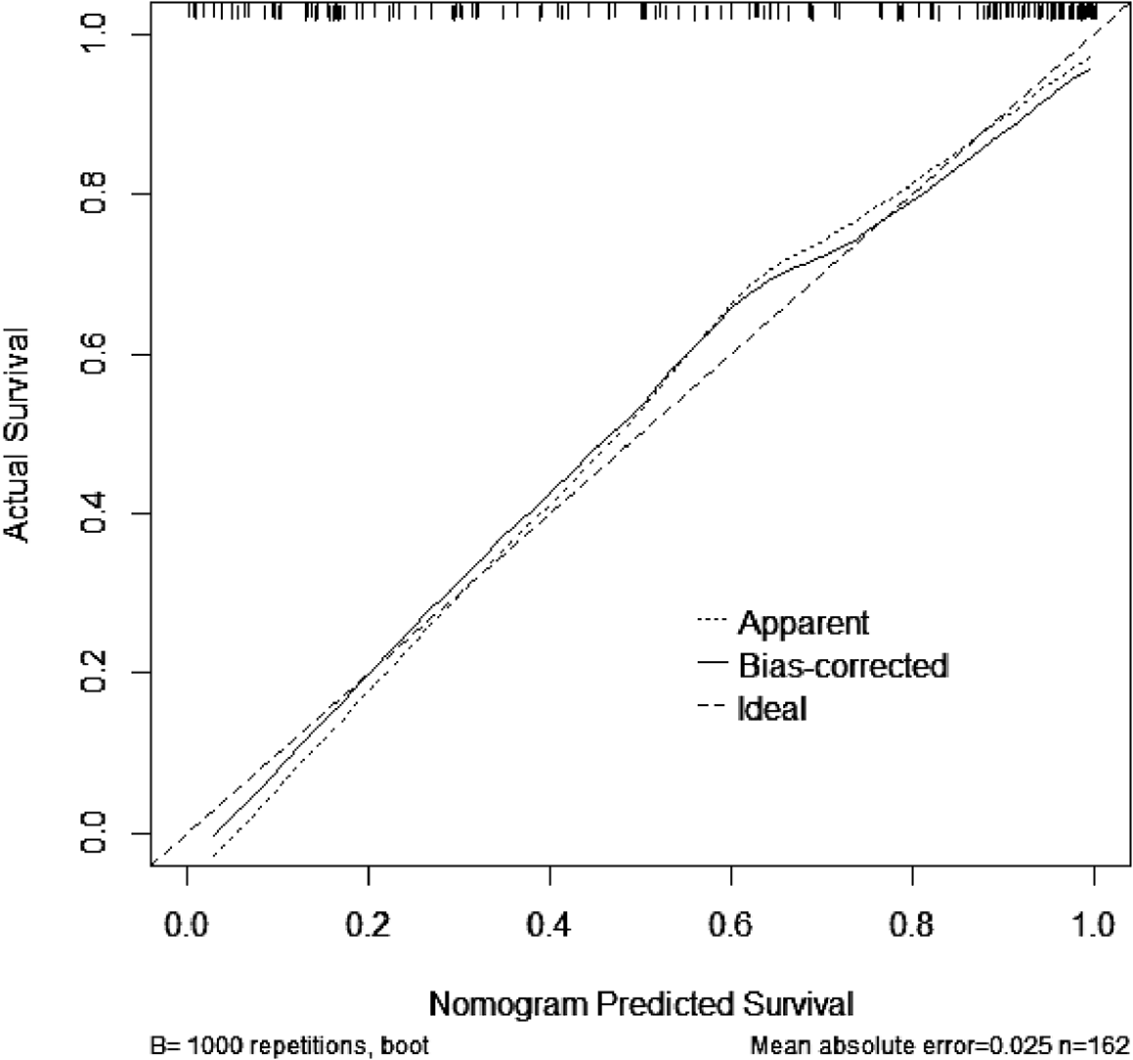
Calibration curve of the LEAD prediction model. The x-axis represents the predicted probability of the model. The y- axis represents the actual probability of occurrence. The diagonal dotted line represents a perfect prediction by an ideal model. The solid line represents the model curve calibrated by 1000 bootstrap resampling methods, a closer fit to the diagonal dotted line represents a better prediction.

Calibration curve: The calibration degree C-index value of the test and evaluation of the nomogram was 0.898. The predicted results of the model were close to reality. The nomogram was consistent in predicting the occurrence of LEAD in patients with T2DM (Fig. 7).

**Fig. 8:**
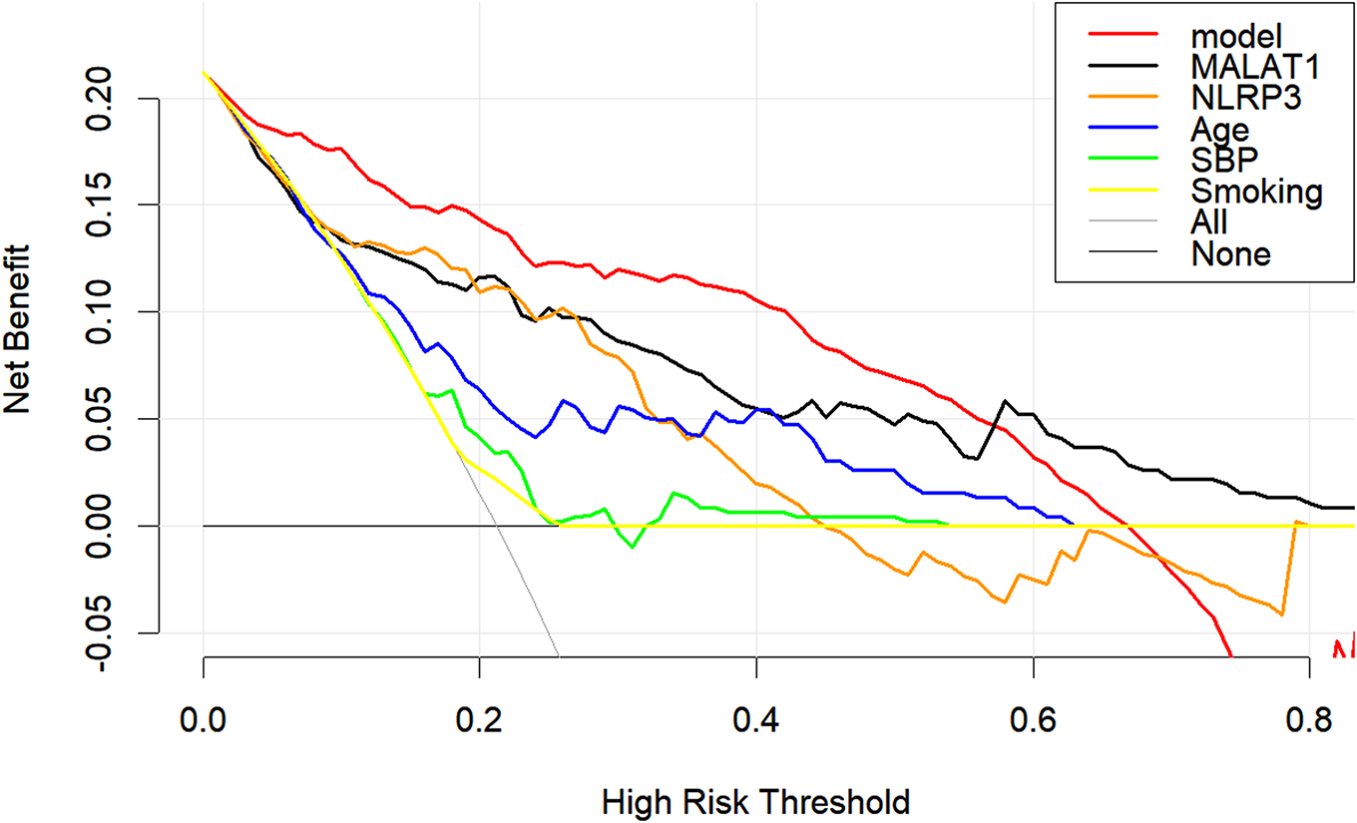
DCA curve of the LEAD prediction model. The x-axis in the figure represents the threshold probability, the y-axis represents the net benefit rate. The horizontal black solid line indicates that all patients did not receive clinical intervention, the gray diagonal line indicates that all patients received clinical intervention, and the colored curve represents the decision curve for each indicator.

Decision curve: The clinical validity of the predictive model was assessed using DCA. When the threshold probabilities were between 0 and 0.58, the net benefit rates obtained from using the predictive model for clinical intervention decision-making were all greater than those obtained from using the separate factors within the model for decision-making. On the other hand, when the threshold probability is between 0 and 0.66, the net benefit rate obtained by using the predictive model is higher than that obtained by not intervening in all patients or intervening in all patients (Fig. 8).

**Table 3:** Value analysis of predicting T2DM + LEAD using relevant indicators

test	AUC	95%CI	Cut-off	specificity	sensitivity	Accuracy	Youden
Age	0.715	0.638–0.793	58.500	0.797	0.541	0.642	1.338
SBP	0.596	0.506–0.686	122.500	0.453	0.745	0.630	1.198
Smoking	0.550	0.473–0.627	1.500	0.641	0.459	0.531	1.100
MALAT1	0.804	0.736–0.872	2.510	0.781	0.765	0.772	1.547
NLRP3	0.794	0.719–0.869	3.310	0.750	0.786	0.772	1.536
Model	0.898	0.847–0.950	0.501	0.797	0.888	0.852	1.685

Model The nomogram for LEAD in patients with T2DM was developed in the cohort by integrating

Age, smoking, SBP, MALAT1, and NLRP3

## Discussion

Atherosclerotic diseases have become the leading cause of death from the disease worldwide, and diabetes is an important risk factor for AS that can accelerate and exacerbate atherosclerosis, a major cause of diabetes morbidity and mortality [13]. LEAD has an insidious onset, lacks typical symptoms, progresses to diabetic foot or even amputation in severe cases, and lacks specific treatment. Prolonged hyperglycemia and insulin resistance promote vascular inflammation, vascular smooth muscle cell growth, dyslipidemia and blood pressure abnormalities, and recruitment of immune cells to the endothelium, leading to vascular endothelial damage [14]. The damage of vascular endothelial cells may lead to extensive pathological changes that cause the development and progression of diabetic vascular complications.

Our results showed that age, disease duration, and the insulin resistance index were greater in the T2DM + LEAD group than in the T2DM alone group, which is consistent with the fact that diabetes combined with atherosclerosis in the lower extremities is more common in older people and that the longer the duration of diabetes, the more pronounced the insulin resistance, and the greater the risk of developing atherosclerosis. However, the glycosylated hemoglobin level in the LEAD group was slightly greater than that in the T2DM group, and the FBG was lower than that in the T2DM group. The reason for this difference was that some of the patients with simple T2DM enrolled in the group had a shorter duration of disease, and had not used hypoglycemic drugs, while most of the LEAD patients had a longer duration of disease and were on hypoglycemic drugs for maintenance treatment. Moreover, fasting blood glucose and glycated hemoglobin are only short-term blood glucose levels, which further suggests that long-term chronic and sustained high blood glucose levels are highly important in patients with T2DM complicated with atherosclerotic disease. Hypertension is a common disease in elderly people. A study in the United States found [15] that there is a strong correlation between systolic blood pressure and peripheral vascular diseases, and SBP variability is an important and powerful predictor of lower limb amputation. High SBP tends to lead to reduced arterial elasticity, damage to the intima, and the formation of atherosclerosis, which is in line with the results of this study.

The lncRNA MALAT1 is located at chromosomal region 11q13.1 in humans and is involved in multiple physiopathological processes, including cancer and diabetic complications. Previous studies have focused on the relationship between MALAT1 and microvascular and coronary heart disease in diabetes. The role of lncRNA MALAT1 in T2DM patients with LEAD has not been reported. Puthanveetil [6] et al. demonstrated for the first time that the expression of MALAT1 is elevated in human umbilical vein endothelial cells after incubation in high glucose and that MALAT1 regulates high glucose-induced inflammatory processes. In vivo studies have shown that MALAT1 expression is increased in the kidneys of diabetic mice. Yan [16] et al. showed that MALAT1 is a conserved lncRNA that may be a potential therapeutic target for the diagnosis and treatment of diabetic retinopathy. Recent studies [8, 17] have shown that MALAT1 expression is significantly elevated in the peripheral blood of patients with coronary artery disease; lncRNA MALAT1 gene expression is upregulated in patients with diabetes mellitus combined with coronary artery disease. Our results demonstrated that the expression level of lncRNA MALAT1 in the LEAD group was significantly greater than that in the simple diabetes group, suggesting that MALAT1 plays a role in diabetic lower limb atherosclerotic disease. The mechanism may be that MALAT1 regulates the inflammatory response of endothelial cells induced by high glucose to participate in the formation of atherosclerotic plaque [6].

Diabetes and AS are closely related to the pathogenesis of inflammatory response and excessive immune activation [18]. The NLRP3 inflammasome is the most studied and is a key mediator of inflammation and immunity. NLRP3 activation recruits to the precursor of cysteine caspase-1 (caspase-1) and triggers auto-protein hydrolysis to generate active caspase-1, leading to cleavage of Gasdermin D and membrane rupture, initiating pyroptosis, and release of inflammatory cytokines [19, 20]. High glucose as a danger-associated molecule activates NLRP3 inflammatory vesicles, triggering an inflammatory response and cellular pyroptosis [21]. Previous studies [22] found that abnormal activation of NLRP3 inflammasome in diabetes patients was associated with atherosclerosis. Animal experiments in a diabetic atherosclerosis mouse model showed that knockdown of NLRP3 inhibited the expression of intima-media adhesion molecules, reduced atherosclerosis, and stabilized atherosclerotic plaques. In a clinical study [23], The expression of NLRP3 mRNA was significantly higher in patients with diabetes combined with carotid atherosclerosis than in patients with T2DM alone, and NLRP3 inflammatory vesicle pathway activity was found to be significantly increased in AS and T2DM patients in the early stages of carotid atherosclerosis. The serum NLRP3 concentration in the LEAD group in this study was significantly greater than that in the T2DM group, suggesting that the development of lower limb atherosclerotic lesions combined with T2DM may be related to the increased expression of NLRP3. T2DM combined with atherosclerosis is a chronic inflammatory reaction in vivo caused by the coexistence of multiple risk factors. Hyperglycemia, as an important risk factor, maybe the reason for the increased expression of NLRP3 in T2DM combined with lower limb atherosclerosis. The specific molecular mechanism needs further study.

LncRNA MALAT1 was found to regulate the expression of NLRP3, and upregulation of MALAT1 exacerbated NLRP3-mediated cellular pyroptosis in diabetic rat atherosclerosis [11]. LncRNA MALAT1 affects NLRP3 expression by competitively binding to miR-22 and promoting high glucose-induced endothelial cellular pyroptosis [12]. Logistic regression analysis revealed that NLRP3 and MALAT1 were independent risk factors for LEAD in patients with type 2 diabetes. Correlation analysis revealed in a positive correlation between MALAT1 and NLRP3 in patients in the LEAD group, confirming the close relationship.

In this study, age, SBP, smoking, lncRNA MALAT1, and NLRP3 were screened as independent risk factors for LEAD by LASSO and logistic regression analyses. Age has an important influence on the development of diabetic peripheral vasculopathy (PAD) in patients with T2DM, and the risk of developing PAD is significantly greater in patients who smoke than in nonsmokers [24, 25]. Previous studies have shown the relationships between lncRNA MALAT1 and NLRP3 and diabetic nephropathy [5], cardiomyopathy [26], retinopathy [16], coronary arteries [8], and carotid arteries [23], and the results revealed that lncRNA MALAT1 may serve as a biological predictor of the above diseases. We used ROC curves to analyze the value of different parameters in the diagnosis of LEAD in patients with T2DM, and lncRNA MALAT1, NLRP3, age, SBP, and smoking all had a statistically significant diagnostic value. Among these parameters, the MALAT1 AUC was the largest (AUC = 0.804), with a high predictive value, and could be considered a novel LEAD biomarker of LEAD.

According to previous studies, the combination of multiple variables may improve the diagnosis [27]. We constructed a nomogram prediction model for risk factors and conducted internal validation of the model. The model had a discrimination AUC of 0.898, sensitivity of 88.8%, and specificity of 79.7%. We found that lncRNA MALAT1 was more diagnostic of LEAD than other individual indicators. Combined analysis of MALAT1, NLRP3, age, SBP, and smoking improved the predictive value of MALAT1. The calibration curves showed good consistency. The decision curve suggested that the predictive model had good clinical validity.

## Conclusions

In this study, we confirmed the differential expression of lncRNA MALAT1 and NLRP3 in T2DM patients with LEAD and T2DM patients alone and found that lncRNA MALAT1 can be used as a potential biomarker for lower limb atherosclerosis in patients with T2DM, and revealed the correlation between lncRNA MALAT1 and NLRP3. However, whether lncRNA MALAT1 participates in the pathogenesis of diabetic lower limb atherosclerosis through NLRP3-mediated cellular pyroptosis needs further study. This study explored and established a concise nomogram model for predicting the risk of LEAD in T2DM patients, and found that the model has high accuracy and predictive ability. In clinical application, medical staff can add the corresponding scores of various risk factors (MALAT1, NLRP3, age, SBP, smoking) in the nomogram, and get the risk of lower limb atherosclerosis in T2DM patients according to the total score, which is helpful for clinical evaluation of the condition according to the individual condition of patients. By controlling blood pressure, quitting smoking, and dynamically monitoring MALAT1 and NLRP3 indicators, T2DM patients can delay the occurrence of lower extremity atherosclerosis, prevent it from developing into lower extremity vascular occlusion or diabetes foot and other serious diseases, improve the prognosis and quality of life of patients, and at the same time, it is convenient for doctors to early identify high-risk patients with diabetes who may have LEAD, and develop diagnosis and treatment measures for them. This study has several limitations, as it was a single-center study with a small sample size, and the actual effectiveness of the nomogram may require further validation. Therefore, large-scale cohort studies and in vitro and in vivo studies are need to elucidate the specific pathogenesis of this disease to provide a basis for prevention and treatment. All case data in this study can be found in Supplementary Material 1.

### Electronic supplementary material

Below is the link to the electronic supplementary material.

Supplementary Material 1

## Data Availability

The datasets generated and/or analyzed during the current study can be available from the corresponding author on reasonable request.
